# Helical tomotherapy for single and multiple liver tumours

**DOI:** 10.1186/1748-717X-5-58

**Published:** 2010-06-24

**Authors:** Tsair-Fwu Lee, Pei-Ju Chao, Fu-Min Fang, Te-Jen Su, Stephen W Leung, Hsuan-Chih Hsu

**Affiliations:** 1 Medical Physics and Informatics Lab. (EE), National Kaohsiung University of Applied Sciences, Kaohsiung, Taiwan; 2 Chang Gung Memorial Hospital-Kaohsiung Medical Centre, Chang Gung University College of Medicine, Kaohsiung, Taiwan; 3 Yuan's General Hospital, Kaohsiung, Taiwan

## Abstract

**Purpose:**

Dosimetric evaluations of single and multiple liver tumours performed using intensity-modulated helical tomotherapy (HT) were quantitatively investigated. Step-and-shoot intensity-modulated radiotherapy (SaS-IMRT) was used as a benchmark.

**Methods:**

Sixteen patients separated into two groups with primary hepatocellular carcinomas or metastatic liver tumours previously treated using SaS-IMRT were examined and re-planned by HT. The dosimetric indices used included the conformity index (*CI*) and homogeneity index (*HI*) for the planned target volume (PTV), max/mean dose, quality index (*QI*), normal tissue complication probability (NTCP), *V*_*30 Gy*_, and *V*_*50% *_for the specified organs at risk (OARs). The monitor units per fraction (MU/fr) and delivery time were also analysed.

**Results:**

For the single tumour group, both planning systems satisfied the required PTV prescription, but no statistical significance was shown by the indexes checking. A shorter delivery time and lower MU/fr value were achieved by the SaS-IMRT. For the group of multiple tumours, the average improvement in *CI *and *HI *was 14% and 4% for HT versus SaS-IMRT, respectively. Lower V_50%_, V_30 Gy _and *QI *values were found, indicating a significant dosimetric gain in HT. The NTCP value of the normal liver was 20.27 ± 13.29% for SaS-IMRT and 2.38 ± 2.25% for HT, indicating fewer tissue complications following HT. The latter also required a shorter delivery time.

**Conclusions:**

Our study suggests dosimetric benefits of HT over SaS-IMRT plans in the case of multiple liver tumours, especially with regards sparing of OARs. No significant dosimetric difference was revealed in the case of single liver tumour, but SaS-IMRT showed better efficiency in terms of MU/fr and delivery time.

## Background

During the past 20 years, primary liver cancer has ranked the fifth most common malignancy worldwide, the third leading cause of death from malignant neoplasm in Japan in men and the fifth in women [1,2], and the second leading cause of cancer death in Taiwan with a mortality of more than 7,000 cases each year [3]. Several modalities have been used for the treatment of hepatocellular carcinomas (HCC) and metastatic liver tumours [4-10] including surgery, transcatheter arterial chemoembolization (TACE), percutaneous ethanol injection therapy, microwave coagulation therapy, radiotherapy and liver transplantation. The role of radiotherapy has been limited because of the poor tolerance of the whole liver to radiation [11,12]. With advances in intensity-modulated radiation therapy (IMRT), several reports have indicated increased safety and more promising results in patients with unresectable intrahepatic malignancies treated with radiotherapy to a portion of the liver [6,13-18]. IMRT constitutes an advanced form of the conformal technique and uses inverse planning algorithms and iterative computer-driven optimization to generate treatment fields with varying beam intensity. It has the ability to produce custom-tailored conformal dose distributions around the tumour, although most studies have examined large tumours [19]. IMRT can also be delivered using linac or Hi-Art Helical Tomotherapy (HT) (TomoTherapy, Madison, WI, USA), which creates a more uniform target dose and improves critical organ sparing [16,20-23] with a greater number of degrees of freedom.

Compared with conventional and other IMRT techniques, HT can potentially produce superior dose distributions (i.e., more uniform dose to the target and lower doses to normal tissues) and is thus being reconsidered for promotion [21,22,24]. In this study, we investigated the potential improvement of HT over step-and-shoot (SaS)-IMRT for the treatment of single or multiple liver tumours. HT plans were compared with IMRT plans for sixteen patients previously treated using SaS-IMRT delivery. The HT plans were designed to emulate as closely as possible the goals and constraints used for SaS-IMRT plans. Dose distributions in the planned target volume (PTV) and organs at risk (OARs) were compared according to the isodose distribution and dose-volume histogram (DVH)-based method using several dosimetric parameters including the conformity index (*CI*) and homogeneity index (*HI*) for the PTV, max/mean dose, quality index (*QI*) for the organs at risk (OARs) [25-29], *V*_*30 Gy*_, *V*_*50%*_, EUD (equivalent uniform dose), and NTCP (normal tissue complication probabilities) for the normal and whole liver. The delivery time and monitor units per fraction (MU/fr) of the two techniques were also compared. SaS-IMRT was used as a benchmark.

## Methods

### Study population

Sixteen consecutive patients (six females, ten males) with primary hepatocellular carcinomas (HCC) or metastatic liver tumours previously treated using SaS-IMRT between March 2006 and March 2008 were examined. The patient characteristics and tumour descriptions are presented in Table 1. The median age was 68 years (range 50-85). Patients were retrospectively grouped to evaluate the influence of the treatment plans. Two groups were formed according to whether they had single (group 1) or multiple (group 2) tumour sites, and interestingly, there were eight in each group. The distributions of clinical stages according to the American Joint Committee on Cancer (AJCC 6^th ^edition) staging system was as follows; I: 1 (6.25%), II: 3 (18.75%), III: 5 (31.25%) and metastasis liver tumour: 7 (43.75%). Six (37.5%) were treated with a combination of chemotherapy.

**Table 1:** Patient characteristics (n = 16)

Characteristics	No. of patients
Age, median years (range)	68 (50-85)
Gender	
Male	10 (62.5%)
Female	6 (37.5%)
Primary HCC (AJCC, 6^th ^edition)	
I	1 (6.25%)
II	3 (18.75%)
III	5 (31.25%)
Metastasis liver tumour Structures (cm^3^) Mean ± SD (range)	7 (43.75%)
PTV	222.77 ± 170.35 (57.75-726.32)
Normal liver	1299.88 ± 279.03 (751.03-1776.16)
Rt kidney	132.7 ± 50.19 (35.39-238.91)
Lt kidney	147.62 ± 42.82 (78.54-233.17)
Spinal cord	14.10 ± 5.52 (4.93-26.44)
Patient's tumour number	
Single (group 1)	8(50%)
Multiple (group 2)	8(50%)

*Abbreviation: *HCC: Hepatocellular Carcinoma; AJCC = American Joint Committee on Cancer; PTV: Planned target volume; Rt: Right side; Lt: Left side;

All patients were immobilized using a tailor-made vacuum lock in the supine position with their arms placed on their forehead. The patients were scanned using a CT (Siemens Biograph LSO PET/CT, PA, USA) with a 3-mm slice thickness, containing 512 × 512 pixels in each slice. The field of view had a mean dimension of 48 cm.

Treatment plans were originally calculated with the ADAC Pinnacle^3^, version 7.4 (ADAC Inc, CA, USA) treatment-planning system (TPS) on a dose grid of 0.4 × 0.4 × 0.3 cm^3 ^without DMPO (direct machine parameter optimization). The 5-field and range 4 × 6 SaS-IMRT technique was used with the dose goal for PTV coverage; initial gantry angles of 20°, 310°, 270°, 220° and 180° were set. The plan was delivered on an Elekta Precise™ Linac equipped with an 80-leaf 1-cm MLC in SaS-IMRT mode. Basically, the IMRT planning system tried to achieve the dose goal target coverage while keeping within the dose constraints of OARs by sequential iteration.

### PTV and normal organ contouring

The planned target volume (PTV) structures were created from the gross tumour volume (GTV) structures. Respiratory motion is the main determinant of PTV expansion. PTVs were based on a 5 mm radial expansion and a 10 mm craniocaudal expansion. Because respiratory motion has been shown to be greater in the craniocaudal dimension than in the anteroposterior and mediolateral dimensions, an asymmetric expansion was used for the PTV [30-33]. The PTV ranged from 57.75 to 726.32 cc (222.77 ± 170.35). For dosimetric analysis, the normal liver volume did not include the PTV. The OARs used in this study were as follows: 1) spinal cord-maximum dose ≤ 45 Gy; 2) kidneys (L & R)-mean dose to bilateral kidneys must be < 16 Gy. If only one kidney is present, not more than 15% of the volume of that kidney can receive ≥ 18 Gy and no more than 30% can receive ≥ 14 Gy; 3) liver-mean liver dose must be ≤ 25 Gy; 4) gastrointestinal system (GIS) (including stomach and small bowels)-maximum dose ≤ 54 Gy; < 10% of each organ volume can receive between 50 and 53.99 Gy, < 15% of the volume of each organ can receive between 45 and 49.99 Gy.

### Treatment plans

In the re-planned HT, three main parameters were selected: the field width (1, 2.5 or 5 cm), pitch (range 0.01-20), and modulation factor (range 1-10). A 2.5-cm field width, a pitch of 0.287 (0.86/3) and a modulation factor of 2 were used in all of the HT plans in this study [34,35]. The software version used for this re-planning study was Hi-Art TomoPlan 2.1 (Tomotherapy Inc., Wisconsin, USA). The selection of these three parameter values was based on preliminary planning exercises that showed them to provide a good balance between ability at dose sculpting and treatment efficiency, in terms of treatment duration and feasibility for routine use. In general, small field dimensions, small pitch and large modulation factors mean longer irradiation times and a better ability for the delivery system to sculpt complex dose distributions with steeper dose gradients [16,21,23,24,36]. For all patients, dose calculation was done on the fine grid, which has a resolution of 1.875 × 1.875 mm^2 ^by the slice thickness of 3 mm for the dose calculation window of 48 × 48 cm^2 ^(256 × 256 pixels). Both planning systems perform iterations during the optimization process. The 0.1 Gy dose bin-size of the dose-volume histograms (DVHs) used in both systems was the same for the subsequent computation of various indices. Plans were run with the goal of delivering the prescribed doses of 60 Gy/30 fractions while meeting the normal tissue constraints for conventional treatment. The PTV doses were prescribed to cover over 95% of the PTV with no greater than a 107% maximum point dose. Having achieved these objectives, the dose plans were made by the same physicist and approved by the same oncologist, who was specialized in liver tumours. The monitor units per fraction (MU/fr), segments and delivery time taken by the two plans were compared. The patient set-up time was not included.

### Plan evaluation

The HT plans were compared with the SaS-IMRT plans using the following dosimetric parameters:

1. ***CI***: a ratio used to evaluate the goodness of fit of the PTV to the prescription isodose volume in the treatment plans:where *V*_*TV *_is the treatment volume of the prescribed isodose lines; *V*_*PTV *_is the volume of the PTV; and *TV*_*PV *_is the volume of *V*_*PTV *_within the *V*_*TV*_. The smaller and closer the value of *CI *is to 1, the better the dose conformity [26,37].

2. ***HI***: a ratio used to evaluate the homogeneity of the PTV. where *D*_5% _and *D*_95 _are the minimum doses delivered to 5% and 95% of the PTV. A larger *HI *indicates poorer homogeneity [38,39].

3. ***QI***: an index used to evaluate the difference in the maximum or mean absorbed dose at serial or parallel OARs, respectively, between HT and SaS-IMRT plans [22,40].

4. *V*_*50%*_: the percentage volume receiving a dose greater than or equal to 50% of the prescribed dose for a normal liver.

5. *V*_*30 Gy*_: the percentage volume receiving a dose greater than or equal to 30 Gy for the whole liver.

6. EUD: equivalent uniform dose, the original definition of the EUD was derived on the basis of a mechanistic formulation using a linear-quadratic cell survival model [41]. Subsequently, Niemierko and Emami suggested a phenomenological model of the form [42]:

where *α *is a unitless model parameter that is specific to the normal structure or tumour of interest, and *ν*_*i *_is unitless and represents the *i*th partial volume receiving dose *D*_*i *_in Gy. Since the relative volume of the whole structure of interest corresponds to 1, the sum of all partial volumes *v*_*i *_will equal 1. For normal tissues, the EUD represents the uniform dose that leads to the same probability of injury as the examined inhomogeneous dose distribution.

7. *NTCP*: an EUD-based normal tissue complication probability (NTCP) was used. Niemierko proposed parameterization of the dose-response characteristics using the logistic function [42,43]:

where *TD*_50 _is the tolerance dose for a 50% complication rate at a specific time interval (e.g., 5 years in the Emami et al. normal tissue tolerance data [44]) when the whole organ of interest is homogeneously irradiated, and *γ*_50 _is a unitless model parameter that is specific to the normal structure and describes the slope of the dose-response curve. Niemierko and Emami suggested that the parameters of *α *and *γ*_50 _should be used in the EUD-based NTCP model. The values of *α*, *γ*_50_, and *TD*_*50 *_used in this study were 3, 3, and 40 Gy respectively, and were based on the Emami data, calculating the BED as 2 Gy/fraction with an *α/β *ratio of 2 [42,44]. The Matlab-2009a software (MathWorks, Inc., Natick, Massachusetts) was used for EUD-based NTCP and CERR (computational environment for radiotherapy research) calculations [45].

### Statistical analyses

The mean values (standard deviation) of the dosimetric data for the sixteen patients were analysed using the paired Wilcoxon signed-rank test to compare the difference between HT and SaS-IMRT. A two-tailed value of *p *< 0.05 was deemed to indicate statistical significance. The SPSS-15.0 software was used for data processing (SPSS, Inc., Chicago, IL).

## Results

### PTV analysis

**Figure 1:**
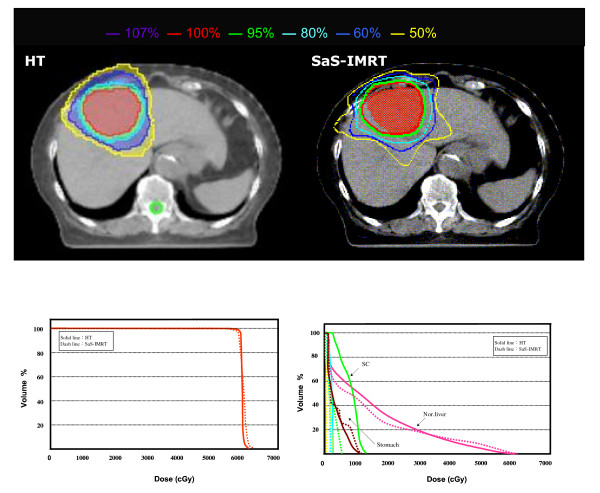
**The comparison of isodose distributions of planned target volume (PTV) and organs at risk (OARs) in an axial plane for one patient in group 1 using the helical tomotherapy (HT) planning system versus step-and-shoot intensity-modulated radiotherapy (SaS-IMRT)**. DVH: Dose volume histograms; PTV = Planning target volume; OAR = Organ at risk

**Figure 2:**
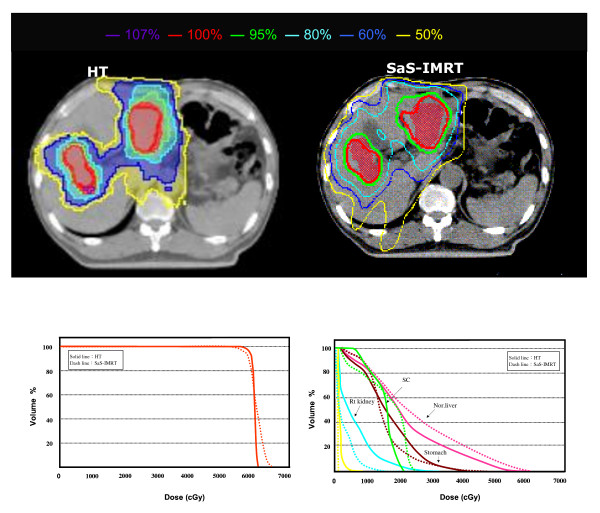
**The comparison of isodose distributions of planned target volume (PTV) and organs at risk (OARs) in an axial plane for one patient in group 2 using the helical tomotherapy (HT) planning system versus step-and-shoot intensity-modulated radiotherapy (SaS-IMRT)**. DVH: Dose volume histograms; PTV = Planning target volume; OAR = Organ at risk

The isodose distributions in the axial plane and the DVHs of the PTV and OARs for one typical case in each group plan using both systems are shown in Figs. 1 and 2.

Table 2 gives the dose statistics for the PTV for each group with HT and SaS-IMRT.

**Table 2:** The dosimetric results of PTV between HT and SaS-IMRT plans for two groups

Parameter	objective	HT	SaS-IMRT	*p*
***Group 1****-single tumour group*				
V_95%_	100	99.44 ± 0.72(97.97-100.00)	99.63 ± 0.51(98.78-100.00)	n/a
V_100%_	95	97.26 ± 1.13(95.87-98.79)	97.84 ± 0.99(96.50-99.07)	n/a
V_107%_	0	0.00 ± 0.00	8.75 ± 4.94(2.30-15.14)	< 0.05
CI	1	1.21 ± 0.07(1.15-1.37)	1.30 ± 0.05(1.23-1.40)	< 0.05
HI	1	1.04 ± 0.01(1.02-1.05)	1.06 ± 0.01(1.02-1.07)	< 0.05
***Group 2****-multiple tumours group*				
V_95%_	100	99.09 ± 0.45(98.43-99.95)	98.47 ± 0.69(97.35-99.25)	n/a
V_100%_	95	96.20 ± 0.70(95.60-97.86)	96.13 ± 1.10(94.80-97.77)	n/a
V_107%_	0	0.30 ± 0.58(0.00-1.60)	16.62 ± 2.38(13.70-20.53)	< 0.05
CI	1	1.25 ± 0.11(1.15-1.47)	1.43 ± 0.07(1.37-1.57)	< 0.05
HI	1	1.06 ± 0.01(1.02-1.07)	1.10 ± 0.02(1.08-1.12)	< 0.05

*Abbreviation: *SaS-IMRT: Step-and-shoot intensity-modulated radiotherapy; HT: Helical tomotherapy; V_x%_: volume receiving ≥x% of the prescribed dose; CI: Conformity index; HI: Homogeneity index; n/a: not statistical significance; statistical significance (p < 0.05) is reported between couples from the paired Wilcoxon signed-rank test analysis.

For group 1, the mean V_95% _and V_100% _for the desired PTV coverage was 99.44 ± 0.72 and 97.26 ± 1.13 in the HT plans, and 99.63 ± 0.51 and 97.84 ± 0.99 in the SaS-IMRT plans, respectively, with no significant differences between plans. For the hot spot checking, the mean V_107% _for the desired PTV was 0.00 ± 0.00 with HT and 8.75 ± 4.94 with SaS-IMRT respectively, indicating significantly better homogeneity of the PTV with HT (p < 0.05). (V_x%_: volume receiving ≥x% of the prescribed dose).

The mean *CI *for group 1 was 1.21 ± 0.07 with HT and 1.30 ± 0.05 with SaS-IMRT, indicating a significantly better conformity of the PTV with HT (p < 0.05) The average improvement in *CI *was 7% for HT. The mean *HI *was 1.04 ± 0.01 for HT and 1.06 ± 0.01 for SaS-IMRT; this difference was statistically significant (*p *< 0.05) with a 2% improvement in HT.

For group 2, the mean V_95% _and V_100% _for the desired PTV coverage was 99.09 ± 0.45 and 96.20 ± 0.70 in the HT plans, and 98.47 ± 0.69 and 96.13 ± 1.10 in the SaS-IMRT plans, respectively, with no significant difference between plans. For the hot spot checking, the mean V_107% _for the desired PTV was 0.30 ± 0.58 with HT and 16.62 ± 2.38 with SaS-IMRT respectively, indicating significantly better homogeneity of the PTV with HT (p < 0.05).

The mean *CI *was 1.25 ± 0.11 with HT and 1.43 ± 0.07 with SaS-IMRT, indicating significantly better conformity of the PTV with HT (p < 0.05). The average improvement in *CI *was 14% for HT versus SaS-IMRT. The mean *HI *for group 2 was 1.06 ± 0.01 for HT and 1.10 ± 0.02 for SaS-IMRT; this difference was statistically significant (*p *< 0.05) with a 4% improvement in HT.

### Dosimetry of OARs

The dose statistics of the specified OARs are summarized in Table 3. For group 1, the mean dose, *V*_*50% *_and NTCP value of the normal liver did not differ significantly between the HT and SaS-IMRT plans (*p *> 0.05). Similarly there was no significant difference between plans in the *V*_*30 Gy *_value of the whole liver (*p *> 0.05) or the max/mean dose of the other four OARs (R/Lt kidneys, GIS, and spinal cord) (*p *> 0.05).

**Table 3:** Dosimetric statistics for the specified OARs

Structure	Parameter	HT	SaS-IMRT	p
***Group 1****-single tumour group*				
Normal liver	Mean (Gy)	18.24 ± 6.73(10.84-31.09)	20.01 ± 7.86 (8.37-31.20)	n/a
	V_50%_(%)	19.17 ± 5.62(10.83-22.50)	22.19 ± 7.13(14.17-31.25)	n/a
	EUD	23.68 ± 5.14(16.60-33.97)	29.11 ± 5.46(21.52-37.14)	< 0.05
	NTCP	1.69 ± 4.30(0.003-12.33)	6.80 ± 9.86(0.06-29.07)	0.051
Whole liver	V_30 Gy_(%)	36.41 ± 14.88(16.45-62.00)	39.44 ± 16.57(16.94-62.06)	n/a
Lt kidney	Mean (Gy)	2.48 ± 2.43 (0.30-6.44)	2.83 ± 3.61(0.17-9.00)	n/a
Rt kidney	Mean (Gy)	4.13 ± 3.09 (0.42-8.03)	5.55 ± 4.55(0.15-10.57)	n/a
GIS	Max (Gy)	30.18 ± 18.17(8.65-52.56)	32.67 ± 17.27(11.77-53.45)	n/a
Spinal cord	Max (Gy)	15.30 ± 9.14(5.12-34.28)	22.05 ± 11.10(4.58-34.78)	n/a
				
***Group 2****-multiple tumours group*				
Normal liver	Mean (Gy)	25.89 ± 3.43(18.89-28.45)	29.73 ± 6.71 (15.54-36.96)	< 0.05
	V_50%_(%)	36.46 ± 4.92(29.17-41.67)	51.74 ± 11.46(37.5-69.17)	< 0.05
	EUD	28.09 ± 3.23(21.95-31.87)	34.68 ± 3.80(27.77-38.43)	< 0.05
	NTCP	2.38 ± 2.25(0.07-6.15)	20.27 ± 13.29(1.23-38.33)	< 0.05
Whole liver	V_30 Gy_(%)	43.91 ± 10.43(23.12-53.42)	55.00 ± 14.28(27.11-74.97)	< 0.05
Lt kidney	Mean (Gy)	4.18 ± 2.94 (0.66-9.21)	2.60 ± 2.03(0.37-6.97)	n/a
Rt kidney	Mean (Gy)	6.11 ± 4.16 (0.99-12.38)	6.45 ± 4.76(0.93-14.58)	n/a
GIS	Max (Gy)	39.59 ± 12.42(21.42-53.20)	42.05 ± 12.36(19.67-52.78)	n/a
Spinal cord	Max (Gy)	18.08 ± 5.38(10.58-28.19)	23.66 ± 8.65(8.96-32.20)	< 0.05

Abbreviation: SaS-IMRT: Step-and-shoot intensity-modulated radiotherapy;; HT: helical tomotherapy; EUD: Equivalent uniform dose; NTCP: Normal tissue complication probability; GIS: Gastrointestinal system (including stomach and small bowels); Lt: left side; Rt: right side; n/a: not statistical significance; statistical significance (p < 0.05) is reported between couples from the paired Wilcoxon signed-rank test analysis.

For group 2, the mean dose, *V*_*50% *_and NTCP value of the normal liver were significantly lower in the HT plans versus the SaS-IMRT plans (*p *< 0.05). The *V*_*50% *_value of the normal liver was 36.46 ± 4.92% for HT and 51.74 ± 11.46% for SaS-IMRT, indicating an approximate reduction of 15% in HT. With regards tissue complications the NTCP value of the normal liver was 2.38 ± 2.25% for HT and 20.27 ± 13.29% for SaS-IMRT, indicating an approximate reduction of 18% in HT (NTCP for liver failure). The *V*_*30 Gy *_value of the whole liver differed significantly between plans (*p *< 0.05). The mean value of *V*_*30 Gy *_for the whole liver was 43.91 ± 10.43% for HT and 55.00 ± 14.28% for SaS-IMRT, indicating an approximate 11% reduction in HT. The max/mean dose of the following three OARs (R/Lt kidneys and GIS) did not differ significantly. The maximum dose of the spinal cord was 18.08 ± 5.38 for HT and 23.55 ± 8.65 Gy for SaS-IMRT. These results indicate a significant dosimetric gain in HT and a reduced dose to sensitive structures.

### QI analysis for the OARs

The *QI *values of the OARs for group 1 and group 2 are listed in Table 4; the kidneys were excluded in the *QI *calculation as the test results did not differ significantly.

**Table 4:** The dosimetric comparisons of QI between HT and SaS-IMRT plans

Variables of OARs	*QI-1*	*QI-2*	*QI-whole cohort*
*QI *of parallel organ			
Normal Liver	0.95 ± 0.20 (0.61-1.30)	0.90 ± 0.14 (0.76-1.21)	0.93 ± 0.17 (0.61-1.30)
*QI *of serial organ			
SC	0.86 ± 0.47 (0.16-1.45)	0.83 ± 0.30 (0.56-1.52)	0.85 ± 0.38 (0.16-1.52)
GIS	0.91 ± 0.23 (0.64-1.36)	0.95 ± 0.12 (0.75-1.11)	0.93 ± 0.18 (0.64-1.36)

*Abbreviation: *HT: Helical tomotherapy; SaS-IMRT: Step-and-shoot intensity-modulated radiotherapy; *QI*: Quality index; QI-1: QI-single tumour group; QI-2: QI-multiple tumours group; SC: Spinal cord; GIS: Gastrointestinal system (including stomach and small bowels);

For group 1, of the two serial OARs, the spinal cord showed the most notable improvement [*QI *= 0.86 ± 0.47] followed by GIS [*QI *= 0.91 ± 0.23], indicating an approximate 14% reduction in maximal dose in the spinal cord and a 9% reduction in the GIS in the HT versus SaS-IMRT plans, respectively (*p *> 0.05). Of the only parallel organ (normal liver) calculated, the *QI*_*Parellel *_was 0.95 ± 0.20, indicating an approximate mean dose reduction of 5% in the normal liver in the HT versus SaS-IMRT plans.

For group 2, of the two serial OARs, the spinal cord showed the most notable improvement [*QI *= 0.83 ± 0.30] followed by the GIS [*QI *= 0.95 ± 0.12], indicating an approximate 17% reduction in maximal dose in the spinal cord and a 5% reduction in the GIS in the HT versus SaS-IMRT plans, respectively. Of the only parallel organ (normal liver) calculated, the *QI*_*Parellel *_was 0.93 ± 0.17, indicating an approximate mean dose reduction of 7% in the normal liver by HT.

For the whole study cohort, of the two serial OARs, the spinal cord showed the most notable improvement [*QI *= 0.85 ± 0.38] followed by the GIS [*QI *= 0.93 ± 0.18], indicating an approximate 15% reduction in maximal dose in the spinal cord and a 7% reduction in the GIS by HT. Of the only parallel (normal liver) organ calculated, the *QI *was 0.93 ± 0.17, indicating an approximate mean dose reduction of 7% in the normal liver by HT.

### MU/fr and delivery time

The MU/fr and delivery time of the sixteen patients with HT versus SaS-IMRT are compared in Table 5. For group 1, the mean delivery time was 4.4 ± 1.4 min (range 2.9-6.3) for HT and 3.3 ± 1.4 min (range 1.9-5.2) for SaS-IMRT, with a significant difference between these values (*p *= 0. 00). The mean MU/fr used was 5135 ± 1678 for HT, which was significantly higher than the mean MU/fr of 343 ± 120 in SaS-IMRT (*p *< 0.05).

**Table 5:** Delivery time and MU/fr used in HT and SaS-IMRT plans

	*Group 1-single tumour group*				*Group 2-multiple tumours group*			
	
	HT		SaS-IMRT		HT		SaS-IMRT	
	
	DT (min)	MU/fr	DT (min)	MU/fr	DT (min)	MU/fr	DT (min)	MU/fr
Mean ± SD	4.4 ± 1.4	5135 ± 1678	3.3 ± 1.4	343 ± 120	4.7 ± 0.8	5529 ± 960	6.2 ± 1.4	461 ± 242
(range)	(2.9-6.3)	(3461-7404)	(1.9-5.2)	(227-577)	(3.3-5.7)	(3904-6704)	(4.8-8.8)	(148-698)

*Abbreviation: *HT: Helical tomotherapy; SaS-IMRT: Step-and-shoot intensity-modulated radiotherapy; DT: delivery time; MU/fr: Monitor units used per fraction; Patient setup time was not included.

For group 2, the mean delivery time was 4.7 ± 0.8 min (range 3.3-5.7) for HT and 6.2 ± 1.4 min (range 4.8-8.8) for SaS-IMRT. A significant difference was observed between these values (*p *< 0.05). The mean MU/fr used was 5529 ± 960 for HT, which was significantly higher than the mean MUs of 461 ± 242 in SaS-IMRT (*p *< 0.05).

## Discussion

The benefits of improved dose homogeneity and better sparing of critical organs in HT compared with conventional linac-based IMRT have been reported in prostate cancer [46], intracranial tumours [24], nasopharyngeal carcinoma [22] and other head and neck cancers [47,48], and breast cancer [13]. However, these benefits of IMRT and HT are generally achieved at the cost of a greater volume of normal tissue in the irradiated volume receiving a low dose [29,49]. In addition, radiotherapy for liver tumours is largely limited by the dose to the surrounding normal tissues, primarily the residual normal liver tissue.

One of the major objectives of this study was to determine the achievable gain of HT in single and multiple liver tumour irradiations against a well-investigated and routinely-used clinical technique, SaS IMRT, delivered in a conventional way with SaS-IMRT planning and an Elekta Precise delivery system. Sixteen cases in two groups were investigated in this study. The HT plans had a slightly significantly better conformity and homogeneity to the PTV than SaS-IMRT plans in the whole cohort. However, the dosimetric advantages of the two plans were inconsistent for individual OARs and other indices.

We demonstrated that HT plans significantly improved the conformity index (improvement ratio: 7 and 14%) and homogeneity index (improvement ratio: 2 and 4%) of the PTV compared with SaS-IMRT plans in group 1 and 2, respectively.

However, the difference between the mean/maximal doses of OARs was not statistically significant in group 1, indicating no difference in OARs sparing. Sparing was found in the normal liver with mean values of *QI-1 *= 0.95 ± 0.20 and *QI-2 *= 0.90 ± 0.14, and in the spinal cord with mean values of *QI-1 *= 0.86 ± 0.47 and *QI-2 *= 0.83 ± 0.30 in group 2, indicating a dosimetric gain in the HT plans.

In *V*_*30 Gy *_and *V*_*50% *_analysis, HT showed a significant dosimetric gain in group 2. The results showed that a better (lower) dose was received in HT than that in group 1; for group 2, the mean value of *V*_*50% *_of the whole liver was 36.46 ± 4.92 for HT and 51.74 ± 11.46 for SaS-IMRT, indicating an approximate reduction of 15.3% in HT. The mean value of *V*_*30 Gy *_of the normal liver was 43.91 ± 10.43 for HT and 55.00 ± 14.28 for SaS-IMRT, indicating an approximate reduction of 11.1% in HT. These results showed a significant dosimetric gain in HT and a reduced mean liver dose.

In clinical practice, the V_50% _(fraction of normal liver treated to at least 50% of the isocentre dose) and the V_30 Gy _(the percentage volume receiving a dose greater than or equal to 30 Gy for the whole liver) are the most commonly used indicators for the dose given. According to the Yonsei University guidelines [50], if the percentage of normal liver volume receiving 50% of the isocentre dose was less than 25%, the total dose was increased to 59.4 Gy; if the percentage was 25% to 49%, the dose was 45 to 54 Gy; if the percentage was 50% to 75%, the dose was 30.6 to 45 Gy, and if the dose was more than 75%, no treatment was administered. They showed that the parameter V_50% _can be divided into four categories and used to predict acceptable liver toxicity. In group 2, the V_50% _value of normal liver was 36.46 ± 4.92% for HT and 51.74 ± 11.46% for SaS-IMRT, indicating an opportunity for dose escalation by HT versus SaS-IMRT plans. The NTCP value of the normal liver was 2.38 ± 2.25% for HT and 20.27 ± 13.29% for SaS-IMRT, indicating that a reduction in tissue complications may be achieved by HT versus SaS-IMRT plans.

Kim et al. demonstrated that the V_30 Gy _appears to be a useful dose-volumetric parameter for predicting the risk of radiation-induced hepatic toxicity (RIHT). In their report, grade 2 or worse RIHT was observed in only 2 out of 85 patients (2.4%) with a whole liver volume receiving 30 Gy (V_30 Gy_, whole liver) of ≤60%, and in 11 out of 20 patients (55.0%) with greater than 60% (*p *< 0.001) [12]. When a lower value of V_50% _and/or V_30 Gy _was accomplished, a higher PTV dose could be given. As a result, a lower V_50% _and/or V_30 Gy _can be achieved with HT for the treatment of multiple liver tumours than with SaS-IMRT. Consequently, a higher dose can be given and a higher response can be achieved when HT is selected.

The overall delivery time and average MU/fr used in the HT plans were significantly higher than for SaS-IMRT plans, which are consistent with the results of several studies [13,22,24,47-49]. The delivery time depended on the limitations of gantry rotation and dose prescription in the HT system, while a speed limitation on gantry rotation exists in the HT system. An interesting result occurred in this study in that a contrary result was found in group 2 due to the geometry of the multiple site distribution. The mean delivery time in group 2 was 4.7 ± 0.8 min (range 3.3-5.7) for HT and 6.2 ± 1.4 min (range 4.8-8.8) for SaS-IMRT. This difference was significant (*p *= 0. 01).

We also found that both planning systems satisfied the required PTV prescription, but that better dose conformity and homogeneity were achieved with the HT compared to SaS-IMRT plans in the two groups. No significant was shown for OARs sparing in group 1, especially if the tumour is leaning against the body surface. As the result, general SaS-IMRT can meet the prescription requirements like the HT did, but shown more efficiency in MU/fr used and delivery time saved than HT in group 1.

We did not aim to perform a strict comparison of the two systems, but to retrospectively evaluate the dosimetric difference for the 16 patients that had been successfully treated with step-and-shoot IMRT and re-planned in a routinely-used helical tomotherapy based upon the same planning CT scan; the dose plans were made by the same physicist and approved by the same oncologist who was specialized in liver tumours. We paid careful attention to reducing biases in this study. However, there are some limitations with regard to our results, and although we used the same resolution, voxel size, and binning of the DVHs in both systems, and the same software (CERR), an intrinsic difference in the calculation algorithms or TPS optimization modules (such as DMPO) might produce different results.

## Conclusions

Our study suggests the dosimetric benefits of HT over SaS-IMRT plans in the group with multiple liver tumours, especially with regards sparing of OARs, as it significantly reduced the V_50% _and V_30 Gy _to the normal liver and whole liver respectively. In addition a reduction in the NTCP value indicates that fewer tissue complications may arise in HT plans. Although there was no significant difference in the group with single liver tumour, IMRT showed better efficiency in terms of the MU/fr and delivery time used.

## Competing interests

The authors declare that they have no competing interests.

## Authors' contributions

TFL and PJC: idea and concept. FMF; TJS and SWL: design and development of study. PJC and HCH: statistical analysis. TFL: writing of manuscript and study coordinator. FMF and HCH: final revision of manuscript. All authors read and approved the final manuscript.
